# Extended thrombotic prophylaxis in COVID-19 early discharge: A retrospective cohort study

**DOI:** 10.1371/journal.pone.0340889

**Published:** 2026-01-30

**Authors:** Sebastiaan T. van’t Hoff, Gea Helfrich, Sanne Boerman, Hans A. Hardeman

**Affiliations:** 1 Department of Pulmonary Medicine, Amphia Hospital, Breda, the Netherlands; 2 Department of Pulmonary Diseases, Maasstad Hospital, Rotterdam, the Netherlands; 3 Department of Pulmonology, St. Antonius Hospital, Nieuwegein, the Netherlands; Ataturk University Faculty of Medicine, TÜRKIYE

## Abstract

**Introduction/Background:**

Due to limits in available staff and space during the COVID-19 pandemic, home monitoring programmes were introduced, reducing strain on resources, and preventing readmissions. Several hospitals included prophylaxis for venous thrombotic events (VTE), as COVID-19 appeared to be thrombogenic. Other hospitals did not, expecting patients to be more mobile while at home. Our aim was to determine whether the administration of nadroparin has led to a difference in VTE occurrence between two groups of previously included patients.

**Materials and methods:**

Retrospective cohort study of two cohorts included in home monitoring with the same protocol, except for nadroparin prophylaxis.

**Results:**

663 patients were analysed in equal groups from two hospitals. No significant difference was found in occurrence of VTE after discharge, readmissions in general or readmissions due to VTE in otherwise comparable groups.

**Discussion:**

As opposed to trials determining thrombotic prophylaxis was of benefit after discharge due to COVID-19, we found no difference between our groups. Our study was retrospective and comprised data compiled over almost two years, which provides a relatively large sample size and overview through different treatment regimes.

**Conclusion:**

For the future, thrombotic prophylaxis for COVID-19 home monitoring might not be indicated and reconsidered for different home monitoring programmes.

## Introduction

In March 2020, when COVID-19 was declared a pandemic, hospitals around the world started to get overrun by patients infected with the then novel and now notorious SARS-CoV-2. Patients had a longer duration of stay compared to other (viral) pneumonias, due to a longer taper of oxygen suppletion [1–4]. Quickly, hospitals ran out of space and staff, and other departments were scaled down as well as face-to-face consults cancelled. Attempting to reduce occupancy and duration of hospital stay, a home monitoring programme with an option for oxygen suppletion was developed in the St. Antonius hospital [1]. Patients were eligible for inclusion when their oxygen requirement decreased to or below 3L/min, they were otherwise clinically improving and were able to enter self-gathered data into a mobile application. They were asked to upload their oxygen saturation, measured via a provided pulse oximeter, temperature, and any respiratory symptoms such as dyspnoea scored on a 0–10 numerical rating scale daily through a home telemonitoring application (Luscii, Utrecht, the Netherlands) on their smartphone or tablet. Submitted data were reviewed by a team of medical students supervised by pulmonologists. No venous thrombotic event (VTE) prophylaxis was prescribed, as patients were considered to be fully ambulatory and thus no longer at risk of developing a VTE. For a more detailed description of the home telemonitoring process we would like to refer to Grutters et al. [1].

After what had become apparent as the first wave of COVID-19 cases, we reported good outcomes using this protocol [1,2,5]. During later waves, over 20 hospitals in the Netherlands and the United Kingdom copied and/or adapted this protocol, adjusting it to their needs as they saw fit. COVID-19 home monitoring has now been proven a safe and effective programme for patients who are asymptomatic, recovering or have mild symptoms. Home monitoring is also already proven in reducing hospital readmissions. [1,6]

As the pandemic progressed, it became apparent that patients with COVID-19 frequently developed thrombotic events, now dubbed CIC (COVID-19 Induced Coagulopathy) [7,8], noting an incidence of VTE as high as 31% and arterial thrombosis of 3.7% [9] in selected cases in the first half of 2020. During that time in autopsies, microthrombotic changes in lung parenchyma were noted in up to 80% of patients [10]. Over the following years, multiple studies have proven benefit in inpatient use of anticoagulation therapy by decreasing COVID-19 mortality, by reducing micro- and macrovascular thrombosis, which thus has become included in Dutch national guidelines for treating inpatient COVID-19 patients [8,11,12]. Part of pre-pandemic regular practice in the Netherlands included routinely prescribing inpatients with a higher risk of VTE antithrombotic prophylaxis, for example risk-guided using the Padua Prediction Score [13]. Only outpatients with longer persisting immobility and at high risk of VTE, such as patients after orthopaedic surgery, were prescribed routine VTE prophylaxis in the home setting.

The Maasstad Ziekenhuis in Rotterdam was the first hospital in the Netherlands that adopted the St. Antonius protocol in 2020. They modified the protocol by prescribing all patients in their home monitoring population a prophylactic dose of anticoagulation (nadroparin, 2850 IE/ 0.3 ml) once daily for the duration of the home monitoring period, as international guidelines and treatment standardization became available and advised VTE prophylaxis, in absence of supporting data [14].

The purpose of this retrospective study is to assess the difference in VTE occurrence between the included groups of the Maasstad and St. Antonius hospitals (hereafter called ‘Rotterdam’ and ‘Nieuwegein’ respectively), as well as evaluate readmission rates with or without VTE. This data can support decision making in the emergent field of early discharge/home monitoring programmes whether to include thromboprophylaxis.

### Design and inclusion

This was a retrospective multicenter database study of all patients enrolled in the COVID-19 home monitoring programmes of both hospitals, starting in April 2020 up until January 2023 for the Nieuwegein population and June 2020 up until January 2023 for Rotterdam. Both hospitals are large teaching hospitals that offered home telemonitoring from an early date in the pandemic. All patients enrolled in the home monitoring programmes consented to their data being used for future research, which was standard for all patients in both hospitals. No ethics board approval was sought for this retrospective data analysis, in concordance with Dutch national ethical guidelines [15]. Data was gathered between June 20^th^ and October 20^th^ 2023 by a team of researchers who weren’t the authors. Thus, the authors had no way of identifying participants.

Both hospitals submitted patient data comprising baseline patient characteristics, oxygen requirement, duration of hospital stay, anticoagulation therapy and 30-day post-discharge occurrence of thrombotic events, such as DVT or PE diagnosed by imaging modalities such as ultrasound or CT angiography, and whether patients had to be readmitted to the hospital.

### Endpoints

We compared patients discharged with prophylactic anticoagulation therapy to patients discharged without. The primary endpoint was the incidence of any venous thrombotic events within 30 days of discharge from the hospital.

Secondary endpoints were the all-cause readmission rate and the readmission rate due to VTE.

### Data collection

Data was collected from EPIC*, Hix 6.1° and Luscii^ by a pool of medical students, already involved as remote monitors in the home monitoring process. Data was cleaned and checked by the primary author for errors and double entries.

* EPIC: Electronic Health Record; Epic, Verona Wisconsin, USA

° Hix 6.1: Electronic Health Record; ChipSoft, Amsterdam, the Netherlands

^ Luscii: home monitoring application for mobile phones or tablets; Luscii, Utrecht, the Netherlands

### Statistical analysis

We used GNU PSPP version 1.2.0 and IBM SPSS statistics version 28.0.1.0 to process our data and run statistical analysis. A chi-square test was run to compare the dichotomous outcomes, while unpaired t-tests were performed for categorical data. For comorbidities, a Fisher’s exact test was performed with variables with less than 5 occurrences. Missing data was subjected to a ‘missing completely at random’ analysis and multiple imputation by expectation maximisation used to supplement data where applicable. A logistic regression analysis was performed to evaluate whether or not VTE rate was related to other covariates. Results were considered statistically significant with an alpha value of p < 0.05.

## Results

Up until January 2023, 666 patients were entered into the database, 337 stemming from Nieuwegein and 329 from Rotterdam. After checking the data and removing double entries, 663 patients were available for analysis on the primary endpoint.

Patient demographics in the included cohorts were comparable, where reported (Table 1). BMI was underreported in enough cases to such degree that multiple imputation could not be used, thus limiting the evaluation of this variable. It was therefore not added to statistical evaluation. Patients in the Nieuwegein group had a higher incidence of pulmonary comorbidities, whereas in the Maasstad group they had a higher incidence of cardiac comorbidity, presumably due to either center having a greater focus on such patient subgroups due to national hospital care centralisation efforts. Overall, other comorbidities between the two groups were similar (Table 2).

**Table 1:** Patient demographics.

*Demographics*	LMWH post discharge (n = 328)	No LMWH post discharge (n = 335)	p-value
*Age (y)*	54.38 [19-85]	56.18 [17-86]	.070
*Sex*	M 189 (57.62%) 17 not registered	M 218 (65.07%)	.258
*Comorbidities*	153	182	.379
*VTE after discharge < 30d*	18 (5.49%)	17 (5.07%)	.812
*Re-admission*	27	28	.953
*Readmission due to VTE*	4	7	.381

^a^Demographics of participants in home monitoring programmes.

**Table 2:** Patient comorbidities.

*Comorbidities*	Nieuwegein (n)	Rotterdam (n)	p-value
*Pulmonary*	95	64	0.008
*Cardiac*	59	78	0.048
*Malignancy*	25	19	0.388
*Renal*	14	19	0.339
*Hepatic*	3	9	0.086
*Neurologic*	29	36	0.315
*Rheumatologic*	15	21	0.274
*Immunocompromised*	17	10	0.152

^a^Comorbidities by organ system.

There was no significant difference in incidence of VTE during admission. On the primary endpoint, a VTE was found in 17 patients (5.07%) in the cohort that did not receive LMWH prophylaxis compared to 18 patients (5.49%) in the group that did receive LMWH. There was no statistically significant difference in all-cause readmission rate, nor in the rate of readmission due to VTE. There is a marked elevation in VTE occurrence in both groups compared to non-COVID pneumonia patients, however (Table 3).

**Table 3:** Endpoint results.

*Occurrence of DVT*	LMWH		Total
	No LMWH	LMWH prophylaxis	
*No DVT*	318	310	628
*Thrombotic event <30 days*	17	18	35
*Total*	335	328	663
*Statistics*	Value	df	Sig
*Pearson’s Chi Square*	.06	1	.812

^a^DVT occurrence by treatment group.

Further logistic regression, after checking for missing values and multiple imputation, showed no statistical difference between groups. Adding comorbidities, age, gender, readmissions for any reason only produced a p = 0.033 for having a male gender, but still no difference for thromboprophylaxis use (Tables 4 and 5).

**Table 4:** Assessing VTE rates between groups.

Classification Table^a^							
	Observed				Predicted		
					DVT		
					No DVT	Thrombotic event <30 days	Percentage Correct
Step 1	VTE	No VTE			628	0	100.0
		thrombotic event <30 days			35	0	.0
	Overall Percentage						94.7
a. The cut value is .500							
**Variables in the Equation**							
		**B**	**S.E.**	**Wald**	**df**	**Sig.**	**Exp(B)**
Step 1^a^	LMWH	.083	.057		1	.812	1.086
	Constant	-2.929	138.427		1	<.001	.053
a. Variable(s) entered on step 1: LMWH.							

Logistic regression result directly comparing thromboprophylaxis versus no thromboprophylaxis group.

**Table 5:** Logistic regression evaluating covariates.

Classification Table^a^							
	Observed				Predicted		
					DVT		
					No DVT	Thrombotic event <30 days	Percentage Correct
Step 1	VTE	No VTE			628	0	100.0
		Thrombotic event <30 days			35	0	.0
	Overall Percentage						94.7
a. The cut value is .500							
**Variables in the Equation**							
		**B**	**S.E.**	**Wald**	**df**	**Sig.**	**Exp(B)**
Step 1^a^	LMWH	.265	.365	.526	1	.468	1.303
	Age	.025	.016	2.550	1	.110	1.026
	Gender	.977	.458	4.554	1	.033	2.656
	Comorbidity	-.279	.723	.149	1	.700	.757
	VTEduring	-.019	11950.767	.000	1	1.000	.981
	Readmission	-18.313	8034.084	.000	1	.998	.000
	ReadmissionVTE	-.275	14753.267	.000	1	1.000	.760
	Lungcomorb	.429	.615	.485	1	.486	1.535
	CARcomorb	.110	.552	.040	1	.842	1.117
	Malignancy	.553	.756	.536	1	.464	1.739
	Nefcomorb	-.492	1.112	.196	1	.658	.612
	Livercomorb	-18.473	11305.107	.000	1	.999	.000
	Neurocomorb	-.576	.805	.513	1	.474	.562
	Reumacomorb	-.503	1.089	.213	1	.644	.605
	Immunocompromised	.243	1.121	.047	1	.829	1.275
	Constant	-5.014	1.056	22.525	1	<.001	.007
a. Variable(s) entered on step 1: LMWH, Age, Gender, Comorbidity, VTEduring, Readmission, ReadmissionVTE, Lungcomorb, CARcomorb, Malignancy, Nefcomorb, Livercomorb, Neurocomorb, Reumacomorb, Immunocompromised.							

Logistic regression result adding age, gender, comorbidities and specified comorbidities, readmission and readmission due to VTE to further identify covariates.

## Discussion

A significant portion of COVID-related morbidity and mortality is thought to be due to coagulopathy [7–11], which might also play a role in long-term complications commonly called long COVID [14,16]. In our cohorts of patients monitored at home with COVID-19, with or without LMWH prophylaxis, we did not find any difference in VTE occurrence or in readmission rate. Earlier studies, such as the RCT by Ramaciotti et al [5], published in 2021 did show a relative risk of 0.33 [0.12–0.90; p = 0.0293] for thrombotic events for patients treated with rivaroxaban discharged after a COVID-19 hospitalization. Their results however have a high chance of selection bias due to 677 of 997 screened patients not being eligible for inclusion, for reasons such as not needing thromboprophylaxis according to current clinical risk scores (n = 175), renal failure (n = 59) or absence of positive PCR (n = 58), amongst others. Of the remaining 320 patients, over 50% (54% in the interventional arm, 50% in the control arm) had an ICU stay during their admission, which carries a higher risk of VTE compared to a normal population [9,17]. A direct oral anticoagulant was not used as thromboprophylaxis in the Netherlands, as it was in the MICHELLE trial [5], because it was not the Dutch standard of care at the time of treatment of this cohort.

We believe our population is a more adequate representation of the general populace, since there were no other eligibility criteria than being currently recovering and being able to use a mobile application. Our data might be a better fit for future patients in general, even though it is a retrospective cohort study which normally have a higher risk of selection bias. By including every patient of the time period we aimed to reduce this risk, though cannot rule out a degree of sampling bias present in all hospital patient population-based research.

A surprising find was the relatively high percentage of VTE occurrence in both our groups, compared to other studies of COVID-19 patients recently described in the meta-analysis by Mehrabi et al. [18], amongst others [19]. However, our higher percentage is in line with the high prevalence of VTE reported in the early stages of the COVID pandemic during admission [7–9], but much higher than reported in large and later series without early discharge or in regular discharged patients with non-COVID pneumonia [18,19]. One possible explanation might be that, due to the earlier discharge from hospital, VTE cases otherwise found during admission [7–9] were now found post-discharge in a variety of time bias. Another explanation might be surveillance bias due to the home monitoring programme and close monitoring of patients during their time in the programme.

Simion et al [20] report a safe use of extended thromboprophylaxis, only finding two VTE occurrences in 177 patients treated with enoxaparin and zero in their 25 patients that were not. However, they excluded 71 patients from the study that received treatment due to a high risk of VTE calculated by Padua Prediction score, possibly introducing exclusion bias.

Courtney et al [21] conclude that post-discharge thromboprophylaxis might be beneficial for patients with COVID-19, based on a cohort of 132 patients that did receive prophylaxis and 1039 that did not. None of the 132 that received prophylaxis had a VTE, while 13 (1.3%) of the other group did (0 vs 1.3%, p = .383), which is a non-significant difference. In our comparatively larger cohorts, there was no difference in incidence between the two groups.

Given that both our groups had the same incidence, we might look at VTEs that originated in hospital and otherwise would have been detected during the admission, despite the normal LMWH prophylaxis. Possible causative factors might include the before mentioned COVID related coagulopathy, but also earlier discharge than in other reports and active surveillance due to the monitoring programme. As not all studies report length of stay, and some have strict inclusion and/or exclusion criteria, direct comparison of our data to the aforementioned studies is not possible. It is encouraging that in our real-world data there was no increased risk in the group without prophylaxis.

Upon reviewing the cases of patients developing a VTE most patients developed such clear symptoms or deterioration that they might have consulted a healthcare professional nonetheless.

It remains unclear why patients in their home situation seem to be less prone to VTE than in a clinical setting. Several factors may be in play, such as decreasing hypercoagulability and inflammation due to natural disease regression, as well as increased mobility. Mobility was however not one of the variables collected in the home monitoring programme. This apparent reduced risk might negate the need for LMWH prophylaxis even in this relatively thrombogenic disease [7,9,10], even though in patients with a post-COVID syndrome (patient dubbed ‘Long COVID’) hypercoagulability has been proven to sometimes persist for months [22]. Due to SARS-CoV-2 mutating and increased immunity in the general population, either through exposure or vaccination, one might consider the possibility of the thrombogenicity of COVID-19 having decreased over time.

Based on our data there seems to be no increased risk of VTE occurrence in patients without extended thromboprophylaxis at home after discharge in a COVID-19 home monitoring programme. Future studies might be necessary to weigh the risk on a per-patient basis, taking into consideration their comorbidities, as well as bleeding risk as several studies covering extended-duration thromboprophylaxis show an increased risk of bleeding events [23].

Strengths of our study are the long observation time, close resemblance to current best practice and a long follow-up of patients. Limitations of our study include it being of a retrospective design which also might encourage missing data, such as BMI. At the time the home monitoring programme in this cohort was used, such programmes were new, privacy concerns not yet fully addressed and biometrics usually specified in prospective trial design not all collected. One mitigation is that our data is otherwise comprised of regularly collected variables in normal practice and thus approximates daily practice.

Home monitoring has proven to be readily available, accessible, and well-received by patients [24]. Home monitoring has seen a boost in use cases since the COVID pandemic and is now being implemented in a wide range of diseases, including early discharge programs. We found no benefit in the use of extended prophylactic LMWH in this relatively thrombogenic disease. Other research groups might reconsider the use of LMWH in their home monitoring protocols, especially when used for less thrombogenic diseases. In the event of a resurgence of COVID-19 or similar disease, a well-defined prospective trial detailing patients to receive thromboprophylaxis of several pharmacological designs compared to placebo should be considered to attempt delivery of a definitive answer on this matter.

## Supporting information

S1 File Original patient database. (SAV)

S2 File Output analysis original patient database. (PDF)

S3 File Logistic regression output original database. (DOCX)

S4 File Missing completely at random (MCAR) analysis. (DOCX)

S5 File Multiple imputation process. (DOCX)

S6 File Database LMWH with imputed data. (XLSX)

S7 File Logistic regression, primary variables, imputed database. (DOCX)

S8 File Logistic regression output all variables, imputed data. (DOCX)
