# Supplementary material for: Extended thrombotic prophylaxis in COVID-19 early discharge: A retrospective cohort study
Source: PLoS One. 2026 Jan 30;21(1):e0340889. doi: 10.1371/journal.pone.0340889 (PMC12857994; doi:10.1371/journal.pone.0340889)
Supplement: S2 File — (PDF) [file pone.0340889.s002.pdf]

```
CROSSTABS /TABLES= LMWH BY      Lungcomorb
/FORMAT=AVALUE TABLES
/STATISTICS=CHISQ
/CELLS=COUNT ROW COLUMN TOTAL.
```

Samenvatting

	Observaties					
	Geldig		Ontbrekend		Totaal	
	N	Procent	N	Procent	N	Procent
LMWH × Lungcomorb	663	100,0%	0	,0%	663	100,0%

LMWH × Lungcomorb

			Lungcomorb		Totaal
			no	yes	
LMWH	No LWMH	Telling	240	95	335
		Rij-%	71,6%	28,4%	100,0%
		Kolom-%	47,6%	59,7%	50,5%
		Totaal-%	36,2%	14,3%	50,5%
	LMWH prophylaxis	Telling	264	64	328
		Rij-%	80,5%	19,5%	100,0%
		Kolom-%	52,4%	40,3%	49,5%
		Totaal-%	39,8%	9,7%	49,5%
Totaal		Telling	504	159	663
		Rij-%	76,0%	24,0%	100,0%
		Kolom-%	100,0%	100,0%	100,0%
		Totaal-%	76,0%	24,0%	100,0%

Chi-kwadraattoetsen

	Waarde	vr.gr	Asympt. sign. (tweezijdig)	Exacte sign. (tweezijdig)	Exacte sign. (éénzijdig)
Pearsons Chi-kwadraat	7,11	1	,008		
Waarschijnlijkheidsratio	7,15	1	,007		
Fishers Exact-toets				,008	,005
Continuïteitscorrectie	6,64	1	,010		
Associatie van lineair met lineair	7,10	1	,008		
N van geldige observaties	663				

```
CROSSTABS /TABLES= Lungcomorb   BY      LMWH
/FORMAT=AVALUE TABLES
/STATISTICS=CHISQ
/CELLS=COUNT ROW COLUMN TOTAL.
```

Samenvatting

	Observaties					
	Geldig		Ontbrekend		Totaal	
	N	Procent	N	Procent	N	Procent
Lungcomorb × LMWH	663	100,0%	0	,0%	663	100,0%

Lungcomorb × LMWH

			LMWH		Totaal
			No LWMH	LMWH prophylaxis	
Lungcomorb	no	Telling	240	264	504
		Rij-%	47,6%	52,4%	100,0%

		LMWH		
		No LWMH	LMWH prophylaxis	Totaal
	Kolom-%	71,6%	80,5%	76,0%
	Totaal-%	36,2%	39,8%	76,0%
yes	Telling	95	64	159
	Rij-%	59,7%	40,3%	100,0%
	Kolom-%	28,4%	19,5%	24,0%
	Totaal-%	14,3%	9,7%	24,0%
Totaal	Telling	335	328	663
	Rij-%	50,5%	49,5%	100,0%
	Kolom-%	100,0%	100,0%	100,0%
	Totaal-%	50,5%	49,5%	100,0%

Chi-kwadraattoetsen

	Waarde	vr.gr	Asympt. sign. (tweezijdig)	Exacte sign. (tweezijdig)	Exacte sign. (éénzijdig)
Pearsons Chi-kwadraat	7,11	1	,008		
Waarschijnlijkheidsratio	7,15	1	,007		
Fishers Exact-toets				,008	,005
Continuïteitscorrectie	6,64	1	,010		
Associatie van lineair met lineair	7,10	1	,008		
N van geldige observaties	663				

```
CROSSTABS /TABLES= LMWH BY      CARcomorb
/FORMAT=AVALUE TABLES
/STATISTICS=CHISQ
/CELLS=COUNT ROW COLUMN TOTAL.
```

Samenvatting

	Observaties					
	Geldig		Ontbrekend		Totaal	
	N	Procent	N	Procent	N	Procent
LMWH × CARcomorb	662	99,8%	1	,2%	663	100,0%

LMWH × CARcomorb

			CARcomorb		
			no	yes	Totaal
LMWH	No LWMH	Telling	276	59	335
		Rij-%	82,4%	17,6%	100,0%
		Kolom-%	52,6%	43,1%	50,6%
		Totaal-%	41,7%	8,9%	50,6%
	LMWH prophylaxis	Telling	249	78	327
		Rij-%	76,1%	23,9%	100,0%
		Kolom-%	47,4%	56,9%	49,4%
		Totaal-%	37,6%	11,8%	49,4%
Totaal		Telling	525	137	662
		Rij-%	79,3%	20,7%	100,0%
		Kolom-%	100,0%	100,0%	100,0%
		Totaal-%	79,3%	20,7%	100,0%

Chi-kwadraattoetsen

	Waarde	vr.gr	Asympt. sign. (tweezijdig)	Exacte sign. (tweezijdig)	Exacte sign. (éénzijdig)
Pearsons Chi-kwadraat	3,93	1	,048		
Waarschijnlijkheidsratio	3,94	1	,047		
Fishers Exact-toets				,055	,030
Continuïteitscorrectie	3,56	1	,059		
Associatie van lineair met lineair	3,92	1	,048		
N van geldige observaties	662				

```
CROSSTABS /TABLES= LMWH BY      Malignancy
/FORMAT=AVALUE TABLES
/STATISTICS=CHISQ
/CELLS=COUNT ROW COLUMN TOTAL.
```

Samenvatting

	Observaties					
	Geldig		Ontbrekend		Totaal	
	N	Procent	N	Procent	N	Procent
LMWH × Malignancy	663	100,0%	0	,0%	663	100,0%

LMWH × Malignancy

			Malignancy		
			no	yes	Totaal
LMWH	No LWMH	Telling	310	25	335
		Rij-%	92,5%	7,5%	100,0%
		Kolom-%	50,1%	56,8%	50,5%
		Totaal-%	46,8%	3,8%	50,5%
	LMWH prophylaxis	Telling	309	19	328
		Rij-%	94,2%	5,8%	100,0%
		Kolom-%	49,9%	43,2%	49,5%
		Totaal-%	46,6%	2,9%	49,5%
Totaal		Telling	619	44	663
		Rij-%	93,4%	6,6%	100,0%
		Kolom-%	100,0%	100,0%	100,0%
		Totaal-%	93,4%	6,6%	100,0%

Chi-kwadraattoetsen

	Waarde	vr.gr	Asympt. sign. (tweezijdig)	Exacte sign. (tweezijdig)	Exacte sign. (éénzijdig)
Pearsons Chi-kwadraat	,75	1	,388		
Waarschijnlijkheidsratio	,75	1	,387		
Fishers Exact-toets				,437	,240
Continuïteitscorrectie	,50	1	,479		
Associatie van lineair met lineair	,74	1	,388		
N van geldige observaties	663				

```
CROSSTABS /TABLES= LMWH BY      Nefcomorb
/FORMAT=AVALUE TABLES
/STATISTICS=CHISQ
/CELLS=COUNT ROW COLUMN TOTAL.
```

Samenvatting

	Observaties					
	Geldig		Ontbrekend		Totaal	
	N	Procent	N	Procent	N	Procent
LMWH × Nefcomorb	663	100,0%	0	,0%	663	100,0%

LMWH × Nefcomorb

			Nefcomorb		Totaal
			no	yes	
LMWH	No LWMH	Telling	321	14	335
		Rij-%	95,8%	4,2%	100,0%
		Kolom-%	51,0%	42,4%	50,5%
		Totaal-%	48,4%	2,1%	50,5%
	LMWH prophylaxis	Telling	309	19	328
		Rij-%	94,2%	5,8%	100,0%
		Kolom-%	49,0%	57,6%	49,5%
		Totaal-%	46,6%	2,9%	49,5%
Totaal		Telling	630	33	663
		Rij-%	95,0%	5,0%	100,0%
		Kolom-%	100,0%	100,0%	100,0%
		Totaal-%	95,0%	5,0%	100,0%

Chi-kwadraattoetsen

	Waarde	vr.gr	Asympt. sign. (tweezijdig)	Exacte sign. (tweezijdig)	Exacte sign. (éénzijdig)
Pearsons Chi-kwadraat	,91	1	,339		
Waarschijnlijkheidsratio	,92	1	,339		
Fishers Exact-toets				,375	,219
Continuïteitscorrectie	,60	1	,437		
Associatie van lineair met lineair	,91	1	,340		
N van geldige observaties	663				

```
CROSSTABS /TABLES= LMWH BY      Livercomorb
/FORMAT=AVALUE TABLES
/STATISTICS=CHISQ
/CELLS=COUNT ROW COLUMN TOTAL.
```

Samenvatting

	Observaties					
	Geldig		Ontbrekend		Totaal	
	N	Procent	N	Procent	N	Procent
LMWH × Livercomorb	663	100,0%	0	,0%	663	100,0%

LMWH × Livercomorb

			Livercomorb		Totaal
			no	yes	
LMWH	No LWMH	Telling	332	3	335
		Rij-%	99,1%	,9%	100,0%
		Kolom-%	51,0%	25,0%	50,5%
		Totaal-%	50,1%	,5%	50,5%
	LMWH prophylaxis	Telling	319	9	328
		Rij-%	97,3%	2,7%	100,0%
		Kolom-%	49,0%	75,0%	49,5%
		Totaal-%	48,1%	1,4%	49,5%

		Livercomorb		Totaal
		no	yes	
Totaal	Telling	651	12	663
	Rij-%	98,2%	1,8%	100,0%
	Kolom-%	100,0%	100,0%	100,0%
	Totaal-%	98,2%	1,8%	100,0%

Chi-kwadraattoetsen

	Waarde	vr.gr	Asympt. sign. (tweezijdig)	Exacte sign. (tweezijdig)	Exacte sign. (éénzijdig)
Pearsons Chi-kwadraat	3,19	1	,074		
Waarschijnlijkheidsratio	3,33	1	,068		
Fishers Exact-toets				,086	,066
Continuïteitscorrectie	2,23	1	,135		
Associatie van lineair met lineair	3,18	1	,074		
N van geldige observaties	663				

```
CROSSTABS /TABLES= LMWH BY      Neurocomorb
/FORMAT=AVALUE TABLES
/STATISTICS=CHISQ
/CELLS=COUNT ROW COLUMN TOTAL.
```

Samenvatting

	Observaties					
	Geldig		Ontbrekend		Totaal	
	N	Procent	N	Procent	N	Procent
LMWH × Neurocomorb	663	100,0%	0	,0%	663	100,0%

LMWH × Neurocomorb

			Neurocomorb		Totaal
			no	yes	
LMWH	No LWMH	Telling	306	29	335
		Rij-%	91,3%	8,7%	100,0%
		Kolom-%	50,6%	50,0%	50,5%
		Totaal-%	46,2%	4,4%	50,5%
	LMWH prophylaxis	Telling	299	29	328
		Rij-%	91,2%	8,8%	100,0%
		Kolom-%	49,4%	50,0%	49,5%
		Totaal-%	45,1%	4,4%	49,5%
Totaal		Telling	605	58	663
		Rij-%	91,3%	8,7%	100,0%
		Kolom-%	100,0%	100,0%	100,0%
		Totaal-%	91,3%	8,7%	100,0%

Chi-kwadraattoetsen

	Waarde	vr.gr	Asympt. sign. (tweezijdig)	Exacte sign. (tweezijdig)	Exacte sign. (éénzijdig)
Pearsons Chi-kwadraat	,01	1	,933		
Waarschijnlijkheidsratio	,01	1	,933		
Fishers Exact-toets				1,000	,521
Continuïteitscorrectie	,00	1	1,000		
Associatie van lineair met lineair	,01	1	,933		

	Waarde	vr.gr	Asympt. sign. (tweezijdig)	Exacte sign. (tweezijdig)	Exacte sign. (éénzijdig)
N van geldige observaties	663				

```
SAVE OUTFILE="C:\Users\b5as4\OneDrive\Bureaublad\AGNES\database 3.sav".

CROSSTABS /TABLES= LMWH BY      Neurocomorb
/FORMAT=AVALUE TABLES
/STATISTICS=CHISQ
/CELLS=COUNT ROW COLUMN TOTAL.
```

Samenvatting

	Observaties					
	Geldig		Ontbrekend		Totaal	
	N	Procent	N	Procent	N	Procent
LMWH × Neurocomorb	663	100,0%	0	,0%	663	100,0%

LMWH × Neurocomorb

			Neurocomorb		Totaal
			no	yes	
LMWH	No LWMH	Telling	306	29	335
		Rij-%	91,3%	8,7%	100,0%
		Kolom-%	51,1%	45,3%	50,5%
		Totaal-%	46,2%	4,4%	50,5%
	LMWH prophylaxis	Telling	293	35	328
		Rij-%	89,3%	10,7%	100,0%
		Kolom-%	48,9%	54,7%	49,5%
		Totaal-%	44,2%	5,3%	49,5%
Totaal		Telling	599	64	663
		Rij-%	90,3%	9,7%	100,0%
		Kolom-%	100,0%	100,0%	100,0%
		Totaal-%	90,3%	9,7%	100,0%

Chi-kwadraattoetsen

	Waarde	vr.gr	Asympt. sign. (tweezijdig)	Exacte sign. (tweezijdig)	Exacte sign. (éénzijdig)
Pearsons Chi-kwadraat	,77	1	,380		
Waarschijnlijkheidsratio	,77	1	,380		
Fishers Exact-toets				,431	,228
Continuïteitscorrectie	,56	1	,455		
Associatie van lineair met lineair	,77	1	,380		
N van geldige observaties	663				

```
SAVE OUTFILE="C:\Users\b5as4\OneDrive\Bureaublad\AGNES\database 3.sav".

CROSSTABS /TABLES= LMWH BY      Neurocomorb
/FORMAT=AVALUE TABLES
/STATISTICS=CHISQ
/CELLS=COUNT ROW COLUMN TOTAL.
```

Samenvatting

	Observaties					
	Geldig		Ontbrekend		Totaal	
	N	Procent	N	Procent	N	Procent
LMWH × Neurocomorb	663	100,0%	0	,0%	663	100,0%

LMWH × Neurocomorb

			Neurocomorb		Totaal
			no	yes	
LMWH	No LWMH	Telling	306	29	335
		Rij-%	91,3%	8,7%	100,0%
		Kolom-%	51,1%	45,3%	50,5%
		Totaal-%	46,2%	4,4%	50,5%
	LMWH prophylaxis	Telling	293	35	328
		Rij-%	89,3%	10,7%	100,0%
		Kolom-%	48,9%	54,7%	49,5%
		Totaal-%	44,2%	5,3%	49,5%
Totaal		Telling	599	64	663
		Rij-%	90,3%	9,7%	100,0%
		Kolom-%	100,0%	100,0%	100,0%
		Totaal-%	90,3%	9,7%	100,0%

Chi-kwadraattoetsen

	Waarde	vr.gr	Asympt. sign. (tweezijdig)	Exacte sign. (tweezijdig)	Exacte sign. (éénzijdig)
Pearsons Chi-kwadraat	,77	1	,380		
Waarschijnlijkheidsratio	,77	1	,380		
Fishers Exact-toets				,431	,228
Continuïteitscorrectie	,56	1	,455		
Associatie van lineair met lineair	,77	1	,380		
N van geldige observaties	663				

```
SAVE OUTFILE="C:\Users\b5as4\OneDrive\Bureaublad\AGNES\database 3.sav".

CROSSTABS /TABLES= LMWH BY      Neurocomorb
/FORMAT=AVALUE TABLES
/STATISTICS=CHISQ
/CELLS=COUNT ROW COLUMN TOTAL.
```

Samenvatting

	Observaties					
	Geldig		Ontbrekend		Totaal	
	N	Procent	N	Procent	N	Procent
LMWH × Neurocomorb	663	100,0%	0	,0%	663	100,0%

LMWH × Neurocomorb

			Neurocomorb		Totaal
			no	yes	
LMWH	No LWMH	Telling	306	29	335
		Rij-%	91,3%	8,7%	100,0%
		Kolom-%	51,2%	44,6%	50,5%
		Totaal-%	46,2%	4,4%	50,5%
	LMWH prophylaxis	Telling	292	36	328
		Rij-%	89,0%	11,0%	100,0%

		Neurocomorb		Totaal
		no	yes	
	Kolom-%	48,8%	55,4%	49,5%
	Totaal-%	44,0%	5,4%	49,5%
Totaal	Telling	598	65	663
	Rij-%	90,2%	9,8%	100,0%
	Kolom-%	100,0%	100,0%	100,0%
	Totaal-%	90,2%	9,8%	100,0%

Chi-kwadraattoetsen

	Waarde	vr.gr	Asympt. sign. (tweezijdig)	Exacte sign. (tweezijdig)	Exacte sign. (éénzijdig)
Pearsons Chi-kwadraat	1,01	1	,315		
Waarschijnlijkheidsratio	1,01	1	,315		
Fishers Exact-toets				,361	,191
Continuïteitscorrectie	,76	1	,383		
Associatie van lineair met lineair	1,01	1	,316		
N van geldige observaties	663				

```
CROSSTABS /TABLES= LMWH BY      Reumacomorb
/FORMAT=AVALUE TABLES
/STATISTICS=CHISQ
/CELLS=COUNT ROW COLUMN TOTAL.
```

Samenvatting

	Observaties					
	Geldig		Ontbrekend		Totaal	
	N	Procent	N	Procent	N	Procent
LMWH × Reumacomorb	663	100,0%	0	,0%	663	100,0%

LMWH × Reumacomorb

			Reumacomorb		Totaal
			no	yes	
LMWH	No LWMH	Telling	320	15	335
		Rij-%	95,5%	4,5%	100,0%
		Kolom-%	51,0%	41,7%	50,5%
		Totaal-%	48,3%	2,3%	50,5%
	LMWH prophylaxis	Telling	307	21	328
		Rij-%	93,6%	6,4%	100,0%
		Kolom-%	49,0%	58,3%	49,5%
		Totaal-%	46,3%	3,2%	49,5%
Totaal		Telling	627	36	663
		Rij-%	94,6%	5,4%	100,0%
		Kolom-%	100,0%	100,0%	100,0%
		Totaal-%	94,6%	5,4%	100,0%

Chi-kwadraattoetsen

	Waarde	vr.gr	Asympt. sign. (tweezijdig)	Exacte sign. (tweezijdig)	Exacte sign. (éénzijdig)
Pearsons Chi-kwadraat	1,20	1	,274		
Waarschijnlijkheidsratio	1,20	1	,273		
Fishers Exact-toets				,306	,178
Continuïteitscorrectie	,85	1	,356		

	Waarde	vr.gr	Asympt. sign. (tweezijdig)	Exacte sign. (tweezijdig)	Exacte sign. (éénzijdig)
Associatie van lineair met lineair	1,19	1	,275		
N van geldige observaties	663				

```
CROSSTABS /TABLES= LMWH BY      Immunocompromised
/FORMAT=AVALUE TABLES
/STATISTICS=CHISQ
/CELLS=COUNT ROW COLUMN TOTAL.
```

Samenvatting

	Observaties					
	Geldig		Ontbrekend		Totaal	
	N	Procent	N	Procent	N	Procent
LMWH × Immunocompromised	663	100,0%	0	,0%	663	100,0%

LMWH × Immunocompromised

			Immunocompromised		Totaal
			no	yes	
LMWH	No LWMH	Telling	325	10	335
		Rij-%	97,0%	3,0%	100,0%
		Kolom-%	51,1%	37,0%	50,5%
		Totaal-%	49,0%	1,5%	50,5%
	LMWH prophylaxis	Telling	311	17	328
		Rij-%	94,8%	5,2%	100,0%
		Kolom-%	48,9%	63,0%	49,5%
		Totaal-%	46,9%	2,6%	49,5%
Totaal		Telling	636	27	663
		Rij-%	95,9%	4,1%	100,0%
		Kolom-%	100,0%	100,0%	100,0%
		Totaal-%	95,9%	4,1%	100,0%

Chi-kwadraattoetsen

	Waarde	vr.gr	Asympt. sign. (tweezijdig)	Exacte sign. (tweezijdig)	Exacte sign. (éénzijdig)
Pearsons Chi-kwadraat	2,05	1	,152		
Waarschijnlijkheidsratio	2,07	1	,150		
Fishers Exact-toets				,172	,108
Continuïteitscorrectie	1,53	1	,217		
Associatie van lineair met lineair	2,05	1	,153		
N van geldige observaties	663				
