# Supplementary material for: Extended thrombotic prophylaxis in COVID-19 early discharge: A retrospective cohort study
Source: PLoS One. 2026 Jan 30;21(1):e0340889. doi: 10.1371/journal.pone.0340889 (PMC12857994; doi:10.1371/journal.pone.0340889)
Supplement: S4 File — (DOCX) [file pone.0340889.s004.docx]

**MVA**

| **Notes** |  |  |
| --- | --- | --- |
| Output Created |  | 08-SEP-2025 22:06:53 |
| Comments |  |  |
| Input | Data | \\storage.erasmusmc.nl\m\MyDocs\106597\My Documents\Desktop\database LMWH.sav |
|  | Active Dataset | DataSet1 |
|  | Filter | <none> |
|  | Weight | <none> |
|  | Split File | <none> |
|  | N of Rows in Working Data File | 663 |
| Syntax |  | MVA VARIABLES=LMWH DVT Age Gender /EM(TOLERANCE=0.001 CONVERGENCE=0.0001 ITERATIONS=25). |
| Resources | Processor Time | 00:00:00.02 |
|  | Elapsed Time | 00:00:00.03 |

| **Univariate Statistics** |  |  |  |  |  |  |  |
| --- | --- | --- | --- | --- | --- | --- | --- |
|  | N | Mean | Std. Deviation | Missing |  | No. of Extremes^a^ |  |
|  |  |  |  | Count | Percent | Low | High |
| LMWH | 663 | .49 | .500 | 0 | .0 | 0 | 0 |
| DVT | 663 | .05 | .224 | 0 | .0 | . | . |
| Age | 646 | 55.31 | 12.622 | 17 | 2.6 | 9 | 0 |
| Gender | 646 | .63 | .483 | 17 | 2.6 | 0 | 0 |

| a. Number of cases outside the range (Q1 - 1.5*IQR, Q3 + 1.5*IQR). |  |  |  |  |  |  |  |
| --- | --- | --- | --- | --- | --- | --- | --- |

| **Summary of Estimated Means** |  |  |  |  |
| --- | --- | --- | --- | --- |
|  | LMWH | DVT | Age | Gender |
| All Values | .49 | .05 | 55.31 | .63 |
| EM | .49 | .05 | 55.38 | .63 |

| **Summary of Estimated Standard Deviations** |  |  |  |  |
| --- | --- | --- | --- | --- |
|  | LMWH | DVT | Age | Gender |
| All Values | .500 | .224 | 12.622 | .483 |
| EM | .500 | .224 | 12.627 | .484 |

**EM Estimated Statistics**

| **EM Means**^a^ |  |  |  |
| --- | --- | --- | --- |
| LMWH | DVT | Age | Gender |
| .49 | .05 | 55.38 | .63 |

| a. Little's MCAR test: Chi-Square = 329.027, DF = 2, Sig. = .000 |  |  |  |
| --- | --- | --- | --- |

| **EM Covariances**^a^ |  |  |  |  |
| --- | --- | --- | --- | --- |
|  | LMWH | DVT | Age | Gender |
| LMWH | .250 |  |  |  |
| DVT | .001 | .050 |  |  |
| Age | -.407 | .173 | 159.443 |  |
| Gender | -.008 | .010 | .151 | .234 |

| a. Little's MCAR test: Chi-Square = 329.027, DF = 2, Sig. = .000 |  |  |  |  |
| --- | --- | --- | --- | --- |

| **EM Correlations**^a^ |  |  |  |  |
| --- | --- | --- | --- | --- |
|  | LMWH | DVT | Age | Gender |
| LMWH | 1 |  |  |  |
| DVT | .009 | 1 |  |  |
| Age | -.064 | .061 | 1 |  |
| Gender | -.034 | .088 | .025 | 1 |

| a. Little's MCAR test: Chi-Square = 329.027, DF = 2, Sig. = .000 |  |  |  |  |
| --- | --- | --- | --- | --- |
