# Supplementary material for: Extended thrombotic prophylaxis in COVID-19 early discharge: A retrospective cohort study
Source: PLoS One. 2026 Jan 30;21(1):e0340889. doi: 10.1371/journal.pone.0340889 (PMC12857994; doi:10.1371/journal.pone.0340889)
Supplement: S5 File — (DOCX) [file pone.0340889.s005.docx]

**MVA**

| **Notes** |  |  |
| --- | --- | --- |
| Output Created |  | 08-SEP-2025 22:09:07 |
| Comments |  |  |
| Input | Data | \\storage.erasmusmc.nl\m\MyDocs\106597\My Documents\Desktop\database LMWH.sav |
|  | Active Dataset | DataSet1 |
|  | Filter | <none> |
|  | Weight | <none> |
|  | Split File | <none> |
|  | N of Rows in Working Data File | 663 |
| Syntax |  | MVA VARIABLES=LMWH DVT Age Gender Comorbidity VTEduring Readmission ReadmissionVTE Lungcomorb CARcomorb Malignancy Nefcomorb Livercomorb Neurocomorb Reumacomorb Immunocompromised /EM(TOLERANCE=0.001 CONVERGENCE=0.0001 ITERATIONS=25 OUTFILE=databaseLMWHimputed). |
| Resources | Processor Time | 00:00:00.11 |
|  | Elapsed Time | 00:00:00.12 |
| Files Saved | EM Imputed Data |  |

| **Univariate Statistics** |  |  |  |  |  |  |
| --- | --- | --- | --- | --- | --- | --- |
|  | N | Mean | Std. Deviation | Missing |  | No. of Extremes^a^ |
|  |  |  |  | Count | Percent | Low |
| LMWH | 663 | .49 | .500 | 0 | .0 | 0 |
| DVT | 663 | .05 | .224 | 0 | .0 | . |
| Age | 646 | 55.31 | 12.622 | 17 | 2.6 | 9 |
| Gender | 646 | .63 | .483 | 17 | 2.6 | 0 |
| Comorbidity | 663 | .53 | .500 | 0 | .0 | 0 |
| VTEduring | 663 | .05 | .211 | 0 | .0 | . |
| Readmission | 663 | .08 | .276 | 0 | .0 | . |
| ReadmissionVTE | 663 | .02 | .128 | 0 | .0 | . |
| Lungcomorb | 663 | .24 | .427 | 0 | .0 | . |
| CARcomorb | 662 | .21 | .405 | 1 | .2 | . |
| Malignancy | 663 | .07 | .249 | 0 | .0 | . |
| Nefcomorb | 663 | .05 | .218 | 0 | .0 | . |
| Livercomorb | 663 | .02 | .133 | 0 | .0 | . |
| Neurocomorb | 663 | .10 | .298 | 0 | .0 | . |
| Reumacomorb | 663 | .05 | .227 | 0 | .0 | . |
| Immunocompromised | 663 | .04 | .198 | 0 | .0 | . |

| **Univariate Statistics** |  |
| --- | --- |
|  | No. of Extremes |
|  | High |
| LMWH | 0 |
| DVT | . |
| Age | 0 |
| Gender | 0 |
| Comorbidity | 0 |
| VTEduring | . |
| Readmission | . |
| ReadmissionVTE | . |
| Lungcomorb | . |
| CARcomorb | . |
| Malignancy | . |
| Nefcomorb | . |
| Livercomorb | . |
| Neurocomorb | . |
| Reumacomorb | . |
| Immunocompromised | . |

|  |  |  |  |  |  |  |
| --- | --- | --- | --- | --- | --- | --- |

| a. Number of cases outside the range (Q1 - 1.5*IQR, Q3 + 1.5*IQR). |  |
| --- | --- |

| **Summary of Estimated Means** |  |  |  |  |  |  |  |  |
| --- | --- | --- | --- | --- | --- | --- | --- | --- |
|  | LMWH | DVT | Age | Gender | Comorbidity | VTEduring | Readmission | ReadmissionVTE |
| All Values | .49 | .05 | 55.31 | .63 | .53 | .05 | .08 | .02 |
| EM | .49 | .05 | 55.35 | .63 | .53 | .05 | .08 | .02 |

| **Summary of Estimated Means** |  |  |  |  |  |  |  |  |
| --- | --- | --- | --- | --- | --- | --- | --- | --- |
|  | Lungcomorb | CARcomorb | Malignancy | Nefcomorb | Livercomorb | Neurocomorb | Reumacomorb | Immunocompromised |
| All Values | .24 | .21 | .07 | .05 | .02 | .10 | .05 | .04 |
| EM | .24 | .21 | .07 | .05 | .02 | .10 | .05 | .04 |

| **Summary of Estimated Standard Deviations** |  |  |  |  |  |  |  |  |
| --- | --- | --- | --- | --- | --- | --- | --- | --- |
|  | LMWH | DVT | Age | Gender | Comorbidity | VTEduring | Readmission | ReadmissionVTE |
| All Values | .500 | .224 | 12.622 | .483 | .500 | .211 | .276 | .128 |
| EM | .500 | .224 | 12.621 | .484 | .500 | .211 | .276 | .128 |

| **Summary of Estimated Standard Deviations** |  |  |  |  |  |  |  |  |
| --- | --- | --- | --- | --- | --- | --- | --- | --- |
|  | Lungcomorb | CARcomorb | Malignancy | Nefcomorb | Livercomorb | Neurocomorb | Reumacomorb | Immunocompromised |
| All Values | .427 | .405 | .249 | .218 | .133 | .298 | .227 | .198 |
| EM | .427 | .405 | .249 | .218 | .133 | .298 | .227 | .198 |

**EM Estimated Statistics**

| **EM Means**^a^ |  |  |  |  |  |  |  |  |
| --- | --- | --- | --- | --- | --- | --- | --- | --- |
| LMWH | DVT | Age | Gender | Comorbidity | VTEduring | Readmission | ReadmissionVTE | Lungcomorb |
| .49 | .05 | 55.35 | .63 | .53 | .05 | .08 | .02 | .24 |

| **EM Means**^a^ |  |  |  |  |  |  |
| --- | --- | --- | --- | --- | --- | --- |
| CARcomorb | Malignancy | Nefcomorb | Livercomorb | Neurocomorb | Reumacomorb | Immunocompromised |
| .21 | .07 | .05 | .02 | .10 | .05 | .04 |

|  |  |  |  |  |  |  |  |  |
| --- | --- | --- | --- | --- | --- | --- | --- | --- |

| a. Little's MCAR test: Chi-Square = 375.209, DF = 29, Sig. = .000 |  |  |  |  |  |  |
| --- | --- | --- | --- | --- | --- | --- |

| **EM Covariances**^a^ |  |  |  |  |  |  |  |
| --- | --- | --- | --- | --- | --- | --- | --- |
|  | LMWH | DVT | Age | Gender | Comorbidity | VTEduring | Readmission |
| LMWH | .250 |  |  |  |  |  |  |
| DVT | .001 | .050 |  |  |  |  |  |
| Age | -.418 | .152 | 159.285 |  |  |  |  |
| Gender | -.008 | .009 | .146 | .234 |  |  |  |
| Comorbidity | -.009 | -.001 | 1.036 | -.009 | .250 |  |  |
| VTEduring | -.007 | -.002 | -.063 | .001 | .000 | .045 |  |
| Readmission | .000 | -.004 | .054 | -.006 | -.003 | .043 | .076 |
| ReadmissionVTE | -.002 | -.001 | .073 | .000 | -.003 | .016 | .015 |
| Lungcomorb | -.022 | .002 | .180 | -.005 | .114 | -.001 | -.003 |
| CARcomorb | .016 | .001 | .752 | .009 | .098 | -.001 | -.002 |
| Malignancy | -.004 | .001 | .356 | -.009 | .031 | .003 | .004 |
| Nefcomorb | .004 | -.001 | .170 | .003 | .024 | .002 | .002 |
| Livercomorb | .005 | -.001 | .081 | .002 | .009 | -.001 | -.002 |
| Neurocomorb | .006 | -.002 | .317 | -.007 | .045 | -.005 | -.001 |
| Reumacomorb | .005 | -.001 | .238 | -.014 | .026 | -.001 | -.003 |
| Immunocompromised | .006 | -.001 | -.033 | -.008 | .019 | .006 | .006 |

| **EM Covariances**^a^ |  |  |  |  |  |  |  |
| --- | --- | --- | --- | --- | --- | --- | --- |
|  | ReadmissionVTE | Lungcomorb | CARcomorb | Malignancy | Nefcomorb | Livercomorb | Neurocomorb |
| LMWH |  |  |  |  |  |  |  |
| DVT |  |  |  |  |  |  |  |
| Age |  |  |  |  |  |  |  |
| Gender |  |  |  |  |  |  |  |
| Comorbidity |  |  |  |  |  |  |  |
| VTEduring |  |  |  |  |  |  |  |
| Readmission |  |  |  |  |  |  |  |
| ReadmissionVTE | .016 |  |  |  |  |  |  |
| Lungcomorb | -.002 | .183 |  |  |  |  |  |
| CARcomorb | .000 | .020 | .164 |  |  |  |  |
| Malignancy | .002 | -.004 | .006 | .062 |  |  |  |
| Nefcomorb | .001 | -.001 | .007 | .000 | .047 |  |  |
| Livercomorb | .000 | -.001 | .002 | -.001 | .002 | .018 |  |
| Neurocomorb | -.002 | -.002 | .005 | .003 | .001 | .001 | .089 |
| Reumacomorb | .001 | -.005 | .004 | .001 | .000 | .001 | .004 |
| Immunocompromised | .002 | -.001 | -.002 | .008 | .007 | .002 | -.001 |

| **EM Covariances**^a^ |  |  |
| --- | --- | --- |
|  | Reumacomorb | Immunocompromised |
| LMWH |  |  |
| DVT |  |  |
| Age |  |  |
| Gender |  |  |
| Comorbidity |  |  |
| VTEduring |  |  |
| Readmission |  |  |
| ReadmissionVTE |  |  |
| Lungcomorb |  |  |
| CARcomorb |  |  |
| Malignancy |  |  |
| Nefcomorb |  |  |
| Livercomorb |  |  |
| Neurocomorb |  |  |
| Reumacomorb | .051 |  |
| Immunocompromised | .004 | .039 |

|  |  |  |  |  |  |  |  |
| --- | --- | --- | --- | --- | --- | --- | --- |

|  |  |  |  |  |  |  |  |
| --- | --- | --- | --- | --- | --- | --- | --- |

| a. Little's MCAR test: Chi-Square = 375.209, DF = 29, Sig. = .000 |  |  |
| --- | --- | --- |

| **EM Correlations**^a^ |  |  |  |  |  |  |  |
| --- | --- | --- | --- | --- | --- | --- | --- |
|  | LMWH | DVT | Age | Gender | Comorbidity | VTEduring | Readmission |
| LMWH | 1 |  |  |  |  |  |  |
| DVT | .009 | 1 |  |  |  |  |  |
| Age | -.066 | .054 | 1 |  |  |  |  |
| Gender | -.035 | .086 | .024 | 1 |  |  |  |
| Comorbidity | -.034 | -.006 | .164 | -.037 | 1 |  |  |
| VTEduring | -.062 | -.052 | -.024 | .005 | -.005 | 1 |  |
| Readmission | -.002 | -.071 | .015 | -.044 | -.021 | .736 | 1 |
| ReadmissionVTE | -.034 | -.031 | .045 | .001 | -.042 | .586 | .432 |
| Lungcomorb | -.104 | .025 | .033 | -.025 | .533 | -.007 | -.028 |
| CARcomorb | .077 | .013 | .147 | .045 | .485 | -.007 | -.019 |
| Malignancy | -.034 | .018 | .113 | -.078 | .253 | .056 | .052 |
| Nefcomorb | .037 | -.023 | .062 | .029 | .217 | .048 | .032 |
| Livercomorb | .069 | -.032 | .048 | .033 | .129 | -.030 | -.041 |
| Neurocomorb | .039 | -.032 | .084 | -.052 | .303 | -.073 | -.007 |
| Reumacomorb | .042 | -.027 | .083 | -.129 | .227 | -.022 | -.048 |
| Immunocompromised | .056 | -.015 | -.013 | -.081 | .195 | .135 | .104 |

| **EM Correlations**^a^ |  |  |  |  |  |  |  |
| --- | --- | --- | --- | --- | --- | --- | --- |
|  | ReadmissionVTE | Lungcomorb | CARcomorb | Malignancy | Nefcomorb | Livercomorb | Neurocomorb |
| LMWH |  |  |  |  |  |  |  |
| DVT |  |  |  |  |  |  |  |
| Age |  |  |  |  |  |  |  |
| Gender |  |  |  |  |  |  |  |
| Comorbidity |  |  |  |  |  |  |  |
| VTEduring |  |  |  |  |  |  |  |
| Readmission |  |  |  |  |  |  |  |
| ReadmissionVTE | 1 |  |  |  |  |  |  |
| Lungcomorb | -.045 | 1 |  |  |  |  |  |
| CARcomorb | -.008 | .114 | 1 |  |  |  |  |
| Malignancy | .060 | -.036 | .058 | 1 |  |  |  |
| Nefcomorb | .025 | -.015 | .076 | -.005 | 1 |  |  |
| Livercomorb | -.018 | -.023 | .042 | -.036 | .073 | 1 |  |
| Neurocomorb | -.043 | -.019 | .044 | .034 | .018 | .031 | 1 |
| Reumacomorb | .021 | -.057 | .042 | .016 | .006 | .017 | .055 |
| Immunocompromised | .093 | -.008 | -.025 | .160 | .163 | .087 | -.017 |

| **EM Correlations**^a^ |  |  |
| --- | --- | --- |
|  | Reumacomorb | Immunocompromised |
| LMWH |  |  |
| DVT |  |  |
| Age |  |  |
| Gender |  |  |
| Comorbidity |  |  |
| VTEduring |  |  |
| Readmission |  |  |
| ReadmissionVTE |  |  |
| Lungcomorb |  |  |
| CARcomorb |  |  |
| Malignancy |  |  |
| Nefcomorb |  |  |
| Livercomorb |  |  |
| Neurocomorb |  |  |
| Reumacomorb | 1 |  |
| Immunocompromised | .085 | 1 |

|  |  |  |  |  |  |  |  |
| --- | --- | --- | --- | --- | --- | --- | --- |

|  |  |  |  |  |  |  |  |
| --- | --- | --- | --- | --- | --- | --- | --- |

| a. Little's MCAR test: Chi-Square = 375.209, DF = 29, Sig. = .000 |  |  |
| --- | --- | --- |
