# Supplementary material for: Extended thrombotic prophylaxis in COVID-19 early discharge: A retrospective cohort study
Source: PLoS One. 2026 Jan 30;21(1):e0340889. doi: 10.1371/journal.pone.0340889 (PMC12857994; doi:10.1371/journal.pone.0340889)
Supplement: S7 File — (DOCX) [file pone.0340889.s007.docx]

**Logistic Regression**

| **Notes** |  |  |
| --- | --- | --- |
| Output Created |  | 08-SEP-2025 22:11:04 |
| Comments |  |  |
| Input | Data | \\storage.erasmusmc.nl\m\MyDocs\106597\My Documents\Bas Antonius\Database LMWH imputed.sav |
|  | Active Dataset | databaseLMWHimputed |
|  | Filter | <none> |
|  | Weight | <none> |
|  | Split File | <none> |
|  | N of Rows in Working Data File | 663 |
| Missing Value Handling | Definition of Missing | User-defined missing values are treated as missing |
| Syntax |  | LOGISTIC REGRESSION VARIABLES DVT /METHOD=ENTER LMWH Age Gender Comorbidity VTEduring Readmission ReadmissionVTE Lungcomorb CARcomorb Malignancy Nefcomorb Livercomorb Neurocomorb Reumacomorb Immunocompromised /CRITERIA=PIN(.05) POUT(.10) ITERATE(20) CUT(.5). |
| Resources | Processor Time | 00:00:00.02 |
|  | Elapsed Time | 00:00:00.02 |

| **Case Processing Summary** |  |  |  |
| --- | --- | --- | --- |
| Unweighted Cases^a^ |  | N | Percent |
| Selected Cases | Included in Analysis | 663 | 100.0 |
|  | Missing Cases | 0 | .0 |
|  | Total | 663 | 100.0 |
| Unselected Cases |  | 0 | .0 |
| Total |  | 663 | 100.0 |

| a. If weight is in effect, see classification table for the total number of cases. |  |  |  |
| --- | --- | --- | --- |

| **Dependent Variable Encoding** |  |
| --- | --- |
| Original Value | Internal Value |
| No DVT | 0 |
| thrombotic event <30 days | 1 |

**Block 0: Beginning Block**

| **Classification Table**^a,b^ |  |  |  |  |  |
| --- | --- | --- | --- | --- | --- |
|  | Observed |  | Predicted |  |  |
|  |  |  | DVT |  | Percentage Correct |
|  |  |  | No DVT | thrombotic event <30 days |  |
| Step 0 | DVT | No DVT | 628 | 0 | 100.0 |
|  |  | thrombotic event <30 days | 35 | 0 | .0 |
|  | Overall Percentage |  |  |  | 94.7 |

| a. Constant is included in the model. |  |  |  |  |  |
| --- | --- | --- | --- | --- | --- |
| b. The cut value is .500 |  |  |  |  |  |

| **Variables in the Equation** |  |  |  |  |  |  |  |
| --- | --- | --- | --- | --- | --- | --- | --- |
|  |  | B | S.E. | Wald | df | Sig. | Exp(B) |
| Step 0 | Constant | -2.887 | .174 | 276.354 | 1 | <.001 | .056 |

| **Variables not in the Equation** |  |  |  |  |  |
| --- | --- | --- | --- | --- | --- |
|  |  |  | Score | df | Sig. |
| Step 0 | Variables | LMWH | .057 | 1 | .812 |
|  |  | Age | 1.996 | 1 | .158 |
|  |  | Gender | 5.158 | 1 | .023 |
|  |  | Comorbidity | .022 | 1 | .883 |
|  |  | VTEduring | 1.812 | 1 | .178 |
|  |  | Readmission | 3.343 | 1 | .068 |
|  |  | ReadmissionVTE | .623 | 1 | .430 |
|  |  | Lungcomorb | .427 | 1 | .513 |
|  |  | CARcomorb | .105 | 1 | .746 |
|  |  | Malignancy | .223 | 1 | .637 |
|  |  | Nefcomorb | .351 | 1 | .553 |
|  |  | Livercomorb | .681 | 1 | .409 |
|  |  | Neurocomorb | .699 | 1 | .403 |
|  |  | Reumacomorb | .476 | 1 | .490 |
|  |  | Immunocompromised | .140 | 1 | .709 |
|  | Overall Statistics |  | 13.684 | 15 | .550 |

**Block 1: Method = Enter**

| **Omnibus Tests of Model Coefficients** |  |  |  |  |
| --- | --- | --- | --- | --- |
|  |  | Chi-square | df | Sig. |
| Step 1 | Step | 18.424 | 15 | .241 |
|  | Block | 18.424 | 15 | .241 |
|  | Model | 18.424 | 15 | .241 |

| **Model Summary** |  |  |  |
| --- | --- | --- | --- |
| Step | -2 Log likelihood | Cox & Snell R Square | Nagelkerke R Square |
| 1 | 255.595^a^ | .027 | .081 |

| a. Estimation terminated at iteration number 20 because maximum iterations has been reached. Final solution cannot be found. |  |  |  |
| --- | --- | --- | --- |

| **Classification Table**^a^ |  |  |  |  |  |
| --- | --- | --- | --- | --- | --- |
|  | Observed |  | Predicted |  |  |
|  |  |  | DVT |  | Percentage Correct |
|  |  |  | No DVT | thrombotic event <30 days |  |
| Step 1 | DVT | No DVT | 628 | 0 | 100.0 |
|  |  | thrombotic event <30 days | 35 | 0 | .0 |
|  | Overall Percentage |  |  |  | 94.7 |

| a. The cut value is .500 |  |  |  |  |  |
| --- | --- | --- | --- | --- | --- |

| **Variables in the Equation** |  |  |  |  |  |  |  |
| --- | --- | --- | --- | --- | --- | --- | --- |
|  |  | B | S.E. | Wald | df | Sig. | Exp(B) |
| Step 1^a^ | LMWH | .265 | .365 | .526 | 1 | .468 | 1.303 |
|  | Age | .025 | .016 | 2.550 | 1 | .110 | 1.026 |
|  | Gender | .977 | .458 | 4.554 | 1 | .033 | 2.656 |
|  | Comorbidity | -.279 | .723 | .149 | 1 | .700 | .757 |
|  | VTEduring | -.019 | 11950.767 | .000 | 1 | 1.000 | .981 |
|  | Readmission | -18.313 | 8034.084 | .000 | 1 | .998 | .000 |
|  | ReadmissionVTE | -.275 | 14753.267 | .000 | 1 | 1.000 | .760 |
|  | Lungcomorb | .429 | .615 | .485 | 1 | .486 | 1.535 |
|  | CARcomorb | .110 | .552 | .040 | 1 | .842 | 1.117 |
|  | Malignancy | .553 | .756 | .536 | 1 | .464 | 1.739 |
|  | Nefcomorb | -.492 | 1.112 | .196 | 1 | .658 | .612 |
|  | Livercomorb | -18.473 | 11305.107 | .000 | 1 | .999 | .000 |
|  | Neurocomorb | -.576 | .805 | .513 | 1 | .474 | .562 |
|  | Reumacomorb | -.503 | 1.089 | .213 | 1 | .644 | .605 |
|  | Immunocompromised | .243 | 1.121 | .047 | 1 | .829 | 1.275 |
|  | Constant | -5.014 | 1.056 | 22.525 | 1 | <.001 | .007 |

| a. Variable(s) entered on step 1: LMWH, Age, Gender, Comorbidity, VTEduring, Readmission, ReadmissionVTE, Lungcomorb, CARcomorb, Malignancy, Nefcomorb, Livercomorb, Neurocomorb, Reumacomorb, Immunocompromised. |  |  |  |  |  |  |  |
| --- | --- | --- | --- | --- | --- | --- | --- |
