# Supplementary material for: Extended thrombotic prophylaxis in COVID-19 early discharge: A retrospective cohort study
Source: PLoS One. 2026 Jan 30;21(1):e0340889. doi: 10.1371/journal.pone.0340889 (PMC12857994; doi:10.1371/journal.pone.0340889)
Supplement: S8 File — (DOCX) [file pone.0340889.s008.docx]

**Logistic Regression**

| **Notes** |  |  |
| --- | --- | --- |
| Output Created |  | 08-SEP-2025 21:53:02 |
| Comments |  |  |
| Input | Data | \\storage.erasmusmc.nl\m\MyDocs\106597\My Documents\Desktop\database LMWH.sav |
|  | Active Dataset | DataSet1 |
|  | Filter | <none> |
|  | Weight | <none> |
|  | Split File | <none> |
|  | N of Rows in Working Data File | 663 |
| Missing Value Handling | Definition of Missing | User-defined missing values are treated as missing |
| Syntax |  | LOGISTIC REGRESSION VARIABLES DVT /METHOD=ENTER LMWH Lungcomorb CARcomorb Malignancy Nefcomorb Livercomorb Neurocomorb Reumacomorb Immunocompromised VTEduring /CRITERIA=PIN(.05) POUT(.10) ITERATE(20) CUT(.5). |
| Resources | Processor Time | 00:00:00.00 |
|  | Elapsed Time | 00:00:00.00 |

| **Case Processing Summary** |  |  |  |
| --- | --- | --- | --- |
| Unweighted Cases^a^ |  | N | Percent |
| Selected Cases | Included in Analysis | 662 | 99.8 |
|  | Missing Cases | 1 | .2 |
|  | Total | 663 | 100.0 |
| Unselected Cases |  | 0 | .0 |
| Total |  | 663 | 100.0 |

| a. If weight is in effect, see classification table for the total number of cases. |  |  |  |
| --- | --- | --- | --- |

| **Dependent Variable Encoding** |  |
| --- | --- |
| Original Value | Internal Value |
| No DVT | 0 |
| thrombotic event <30 days | 1 |

**Block 0: Beginning Block**

| **Classification Table**^a,b^ |  |  |  |  |  |
| --- | --- | --- | --- | --- | --- |
|  | Observed |  | Predicted |  |  |
|  |  |  | DVT |  | Percentage Correct |
|  |  |  | No DVT | thrombotic event <30 days |  |
| Step 0 | DVT | No DVT | 627 | 0 | 100.0 |
|  |  | thrombotic event <30 days | 35 | 0 | .0 |
|  | Overall Percentage |  |  |  | 94.7 |

| a. Constant is included in the model. |  |  |  |  |  |
| --- | --- | --- | --- | --- | --- |
| b. The cut value is .500 |  |  |  |  |  |

| **Variables in the Equation** |  |  |  |  |  |  |  |
| --- | --- | --- | --- | --- | --- | --- | --- |
|  |  | B | S.E. | Wald | df | Sig. | Exp(B) |
| Step 0 | Constant | -2.886 | .174 | 276.026 | 1 | <.001 | .056 |

| **Variables not in the Equation** |  |  |  |  |  |
| --- | --- | --- | --- | --- | --- |
|  |  |  | Score | df | Sig. |
| Step 0 | Variables | LMWH | .061 | 1 | .805 |
|  |  | Lungcomorb | .420 | 1 | .517 |
|  |  | CARcomorb | .105 | 1 | .746 |
|  |  | Malignancy | .221 | 1 | .639 |
|  |  | Nefcomorb | .314 | 1 | .575 |
|  |  | Livercomorb | .682 | 1 | .409 |
|  |  | Neurocomorb | .703 | 1 | .402 |
|  |  | Reumacomorb | .479 | 1 | .489 |
|  |  | Immunocompromised | .112 | 1 | .738 |
|  |  | VTEduring | 1.815 | 1 | .178 |
|  | Overall Statistics |  | 4.894 | 10 | .898 |

**Block 1: Method = Enter**

| **Omnibus Tests of Model Coefficients** |  |  |  |  |
| --- | --- | --- | --- | --- |
|  |  | Chi-square | df | Sig. |
| Step 1 | Step | 7.426 | 10 | .685 |
|  | Block | 7.426 | 10 | .685 |
|  | Model | 7.426 | 10 | .685 |

| **Model Summary** |  |  |  |
| --- | --- | --- | --- |
| Step | -2 Log likelihood | Cox & Snell R Square | Nagelkerke R Square |
| 1 | 266.484^a^ | .011 | .033 |

| a. Estimation terminated at iteration number 20 because maximum iterations has been reached. Final solution cannot be found. |  |  |  |
| --- | --- | --- | --- |

| **Classification Table**^a^ |  |  |  |  |  |
| --- | --- | --- | --- | --- | --- |
|  | Observed |  | Predicted |  |  |
|  |  |  | DVT |  | Percentage Correct |
|  |  |  | No DVT | thrombotic event <30 days |  |
| Step 1 | DVT | No DVT | 627 | 0 | 100.0 |
|  |  | thrombotic event <30 days | 35 | 0 | .0 |
|  | Overall Percentage |  |  |  | 94.7 |

| a. The cut value is .500 |  |  |  |  |  |
| --- | --- | --- | --- | --- | --- |

| **Variables in the Equation** |  |  |  |  |  |  |  |
| --- | --- | --- | --- | --- | --- | --- | --- |
|  |  | B | S.E. | Wald | df | Sig. | Exp(B) |
| Step 1^a^ | LMWH | .126 | .353 | .127 | 1 | .721 | 1.134 |
|  | Lungcomorb | .224 | .395 | .322 | 1 | .570 | 1.251 |
|  | CARcomorb | .149 | .424 | .124 | 1 | .725 | 1.161 |
|  | Malignancy | .397 | .647 | .377 | 1 | .539 | 1.487 |
|  | Nefcomorb | -.426 | 1.042 | .167 | 1 | .683 | .653 |
|  | Livercomorb | -18.303 | 11467.499 | .000 | 1 | .999 | .000 |
|  | Neurocomorb | -.667 | .744 | .804 | 1 | .370 | .513 |
|  | Reumacomorb | -.695 | 1.035 | .451 | 1 | .502 | .499 |
|  | Immunocompromised | -.138 | 1.077 | .016 | 1 | .898 | .871 |
|  | VTEduring | -18.416 | 7192.762 | .000 | 1 | .998 | .000 |
|  | Constant | -2.898 | .302 | 92.272 | 1 | <.001 | .055 |

| a. Variable(s) entered on step 1: LMWH, Lungcomorb, CARcomorb, Malignancy, Nefcomorb, Livercomorb, Neurocomorb, Reumacomorb, Immunocompromised, VTEduring. |  |  |  |  |  |  |  |
| --- | --- | --- | --- | --- | --- | --- | --- |
